# Development of a Three-Dimensional Nanostructure SnO_2_-Based Gas Sensor for Room-Temperature Hydrogen Detection

**DOI:** 10.3390/s25154784

**Published:** 2025-08-03

**Authors:** Zhilong Song, Yi Tian, Yue Kang, Jia Yan

**Affiliations:** 1 Institute for Energy Research, School of Future Technology, Jiangsu University, Zhenjiang 212013, China; 2222493029@stmail.ujs.edu.cn (Y.T.); 2222493009@stmail.ujs.edu.cn (Y.K.); 2 Ai-Sensing Technology Co., Ltd., Foshan 528000, China

**Keywords:** gas sensor, SnO_2_, 3D nanostructures, room-temperature, hydrogen

## Abstract

The development of gas sensors with high sensitivity and low operating temperatures is essential for practical applications in environmental monitoring and industrial safety. SnO_2_-based gas sensors, despite their widespread use, often suffer from high working temperatures and limited sensitivity to H_2_ gas, which presents significant challenges for their performance and application. This study addresses these issues by introducing a novel SnO_2_-based sensor featuring a three-dimensional (3D) nanostructure, designed to enhance sensitivity and allow for room-temperature operation. This work lies in the use of a 3D anodic aluminum oxide (AAO) template to deposit SnO_2_ nanoparticles through ultrasonic spray pyrolysis, followed by modification with platinum (Pt) nanoparticles to further enhance the sensor’s response. The as-prepared sensors were extensively characterized, and their H_2_ sensing performance was evaluated. The results show that the 3D nanostructure provides a uniform and dense distribution of SnO_2_ nanoparticles, which significantly improves the sensor’s sensitivity and repeatability, especially in H_2_ detection at room temperature. This work demonstrates the potential of utilizing 3D nanostructures to overcome the traditional limitations of SnO_2_-based sensors.

## 1. Introduction

Hydrogen (H_2_) is considered one of the most promising clean energy sources for the future. Due to its high combustion efficiency and abundance, it plays a pivotal role in achieving carbon neutrality [1,2]. However, the widespread adoption of H_2_ presents significant safety concerns. H_2_ is highly flammable, with a wide flammability range in the air, from as low as 4% to as high as 75% [3]. This makes H_2_ highly susceptible to explosions when within this range. Therefore, it is essential to develop high-performance sensors capable of detecting H_2_ concentrations below 4% for early leak detection. Furthermore, H_2_ molecules are extremely small (with a molar mass of only 2.02 g/mol), enabling them to escape even through tiny cracks in storage tanks, which can occur at concentrations as low as part-per-billion (ppb) levels. This exacerbates the risks of leakage and could lead to material embrittlement or delayed cracking in H_2_ storage systems [4]. Therefore, creating gas sensors with high sensitivity and a broad detection range [5,6,7,8,9,10] is crucial for ensuring the safe use of H_2_ in industrial applications and energy systems.

Semiconductor gas sensors, particularly metal oxide-based sensors, are widely recognized for their high sensitivity to low-concentration gases and fast response times, making them ideal for detecting target gases such as H_2_ [11,12]. Metal oxide sensors, such as those made from SnO_2_, are chemically stable and capable of maintaining long-term operational stability even at elevated temperatures, which makes them suitable for harsh industrial environments [13,14,15]. Studies, such as those by Lv et al., have shown that metal oxide sensors can detect a range of gases, including combustible gases, toxic gases, and volatile organic compounds, through material modifications such as doping and composite structures [16]. However, a significant limitation of most metal oxide gas sensors is their requirement for high operating temperatures (250–500 °C) to activate the surface gas-sensing reactions. This high temperature leads to increased energy consumption and necessitates the use of micro-heaters, which complicates the system and increases its cost [17].

To address these limitations, three-dimensional (3D) nanostructured materials have gained considerable attention for their promising potential in gas-sensor applications. These 3D materials offer exceptional advantages, including extremely low power consumption and superior sensing performance. The high surface-to-volume ratio and interconnected porous structure of 3D nanomaterials provide efficient gas diffusion pathways and an abundance of surface-active sites. This enables highly effective gas detection at room temperature, significantly reducing the energy requirements for gas adsorption and activation [18].

Inspired by these advancements, we present a novel approach, named ultrasonic spray pyrolysis (USP), for fabricating 3D nanostructured gas sensors [19,20]. Specifically, we utilize 3D self-supported nanoporous anodic aluminum oxide (AAO) as the substrate, constructing the sensing layer via USP to ensure uniform and conformal deposition. Initially, a thin SnO_2_ layer is coated onto the substrate, followed by the deposition of platinum (Pt) nanoparticles to enhance the sensor’s response. This USP process enables the creation of a sensor capable of operating at room temperature with a detection limit as low as 285 ppb. Our research emphasizes materials and device engineering, with a focus on the structural, compositional, and morphological characterization of the sensing materials, as well as the evaluation of their H_2_ sensing performance and underlying mechanisms. Through this integrated approach, we achieve low-power, highly sensitive gas detection by leveraging the precision of USP technology. The novelty of this work lies in the development of a 3D nanostructured Pt/SnO_2_ gas sensor through USP on a 3D AAO template. Unlike traditional SnO_2_-based sensors, which typically require high operating temperatures for effective gas sensing, this design enables room-temperature H_2_ detection. The USP process, conducted at 350 °C, results in the uniform and dense distribution of SnO_2_ nanoparticles on the AAO substrate, effectively balancing the porosity for enhanced gas diffusion and ensuring structural stability. The integration of Pt nanoparticles improves the catalytic activity of the sensor, significantly reducing the activation energy for H_2_ detection. This unique combination results in a gas sensor with high sensitivity (detection limit of 285 ppb), a broad detection range (100–10,000 ppm), excellent repeatability, and enhanced humidity tolerance. These attributes address the limitations of conventional sensors, which often suffer from high operating temperatures, low sensitivity, and poor stability. This integrated approach provides a low-power, highly sensitive solution for H_2_ detection.

## 2. Materials and Methods

### 2.1. Fabrication of Pt/SnO_2_ Sensor

SnO_2_ deposition on AAO substrate: A 0.2 mol/L SnCl_4_·5H_2_O ethanol solution was prepared as the precursor for the USP process. The precursor solution was first converted into a vapor phase using an ultrasonic atomizer. The vapor was then carried into a nozzle by controlled dry air. The 3D nanoporous AAO substrate, positioned inside the nozzle, was heated to a temperature range of 310–370 °C for the subsequent USP process. Due to the open-ended structure of the AAO template, the spray pyrolysis process was conducted for 15 min on each side of the AAO substrate.

**Figure 1:**
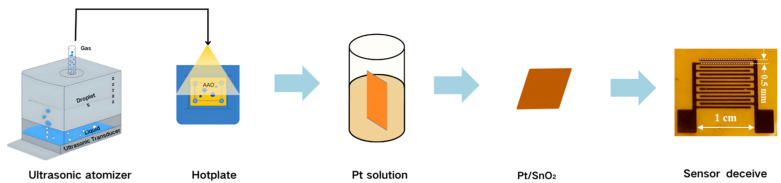
The schematic diagram of Pt/SnO_2_ sensor fabrication.

Metal decoration and gas-sensor fabrication: Following the USP process, the 3D SnO_2_ nanotube structures on the AAO template were immersed in a solution containing platinum (Pt) nanoparticles. Continuous stirring was maintained for 6 h to ensure the uniform deposition of the Pt nanoparticles on the SnO_2_ nanotubes. After this step, the 3D Pt/SnO_2_ nanotube structures were placed under a shadow mask (interdigital space is set at 500 μm, as shown in Figure 1). Subsequently, a 150 nm thick gold (Au) layer was thermally evaporated to form the interdigital electrodes of the gas sensor (the real device is seen in Figure 1).

### 2.2. Characterization

The morphology of the 3D nanostructured SnO_2_ films was examined using high-resolution transmission electron microscopy (HRTEM, JEM 2010F, JEOL, Tokyo, Japan) and scanning electron microscopy (SEM, JSM-7100F, JEOL, Tokyo, Japan). X-ray diffraction (XRD) data were acquired using an X’Pert Pro system, Almelo, The Netherlands, with measurements taken over the 2θ range of 10° to 80°.

### 2.3. Gas Sensing Measurements

The measurement chamber had external dimensions of 160 mm (L) × 90 mm (W) × 52 mm (H), with an internal volume of 75 mL. The sensor was positioned such that its two electrodes contact the measurement probes. The sensor’s resistance was monitored in real time as hydrogen gas (H_2_ 1%, Ar balanced) was introduced into the system via a mass flow controller (MFC). The gas was then mixed with air to achieve the desired target concentration. During the recovery phase, air was flowed through the chamber at a rate of 500 sccm. The sensor’s resistance was measured using an electrochemical workstation. The sensitivity (S) of the gas sensor was defined as follows: $S\;=\;(R_{a}-R_{g})/R_{a}\times 100\%$, where $R_{a}$ represents the initial resistance of the gas sensor in air, and $R_{g}$ is the sensor resistance when exposed to H_2_ gas.

## 3. Results and Discussion

### 3.1. Fabrication and Characterization of the Sensors

As illustrated in Figure 1, the fabrication process of the Pt/SnO_2_ sensor begins with the preparation of a 0.2 mol/L ethanol solution of SnCl_4_·5H_2_O, which is used as the precursor for the USP process. The precursor was atomized into a vapor phase within a containment vessel using an ultrasonic nebulizer, after which the vaporized precursor was transported into a nozzle via a precisely controlled dry air flow. The AAO template positioned within the nozzle was heated to a temperature range of 310–370 °C to facilitate the subsequent spray pyrolysis reaction. Due to the open-ended architecture of the AAO template, a spray pyrolysis treatment with a duration of T = 15 min was performed on each face of the template. This step ensures a uniform deposition of the SnO_2_ nanoparticles on the surface of the AAO template, thereby laying the foundation for the creation of the 3D SnO_2_ nanostructure. Following the spray pyrolysis step, the resulting 3D SnO_2_ nanotube structures on the AAO template were immersed in a solution containing platinum (Pt) nanoparticles. The solution was continuously stirred for 6 h to ensure the uniform deposition of Pt nanoparticles onto the SnO_2_ nanotubes. This step significantly enhances the sensor’s response, providing catalytic properties for H_2_ detection. Once the Pt deposition was complete, the 3D Pt/SnO_2_ nanotube structures were carefully placed under a shadow mask, and subsequently, a 150 nm thick gold (Au) layer was thermally evaporated onto the sensor structure to form the interdigital electrodes. Through these sequential processes, the Pt/SnO_2_ sensor with a three-dimensional nanostructure was fabricated.

**Figure 2:**
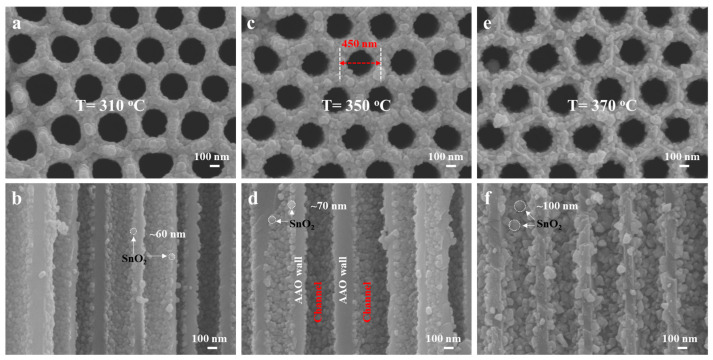
SEM image of SnO_2_ deposited on AAO substrate at temperature of (**a**,**b**) 310 °C; (**c**,**d**) 350 °C; (**e**,**f**) 370 °C.

Figure 2 depicts SEM images of SnO_2_ nanostructures deposited on AAO templates via USP at various temperatures, allowing for a systematic evaluation of thermal effects on morphological evolution and corresponding physicochemical properties. At 310 °C, the surface morphology (Figure 2a) and cross-sectional view (Figure 2b) reveal uniformly distributed SnO_2_ nanoparticles with an average diameter of 60 nm, interspersed with conspicuous interparticle voids. These structural features elongate electron transport pathways, leading to elevated electrical resistance, while the porous architecture would enhance moisture adsorption, rendering the sensor highly susceptible to humidity-induced performance fluctuations, as further discussed later. The underlying mechanism involves competitive adsorption between water molecules and oxygen species on the SnO_2_ surface. At high humidity, water molecules (H_2_O) physically adsorb on the porous SnO_2_ surface via hydrogen bonding, forming a continuous water layer that blocks active sites for oxygen adsorption. This reduces the density of adsorbed oxygen, narrowing the depletion layer of SnO_2_ and decreasing baseline resistance. Additionally, the polar water molecules can capture free electrons from SnO_2_, creating local charge centers that scatter electron transport and exacerbating resistance fluctuations. The loose particle packing further allows water vapor to penetrate deep into the nanostructure, prolonging desorption kinetics and causing persistent resistance drifts with humidity changes. Increasing the deposition temperature to 350 °C yields a denser packing of SnO_2_ nanoparticles (70 nm), as observed in Figure 2c,d. The optimized particle arrangement minimizes interparticle spacing while preserving partial AAO pore connectivity, establishing a critical balance between electrical conductivity and humidity interference. This configuration facilitates moderate electron transport, yielding a stable resistance profile suitable for gas sensing [21]. At 370 °C, SEM analysis (Figure 2e,f) demonstrates significant particle agglomeration, with SnO_2_ nanoparticles reaching 100 nm in size and partially occluding AAO nanopores. While the improved particle connectivity reduces electrical resistance by enhancing electron conduction, the blocked pores hinder the speed of gas diffusion and create areas where moisture can easily accumulate. These changes are likely to affect the sensor’s performance, especially under varying humidity conditions, but specific discussions on these effects can be found in the later sections.

**Figure 3:**
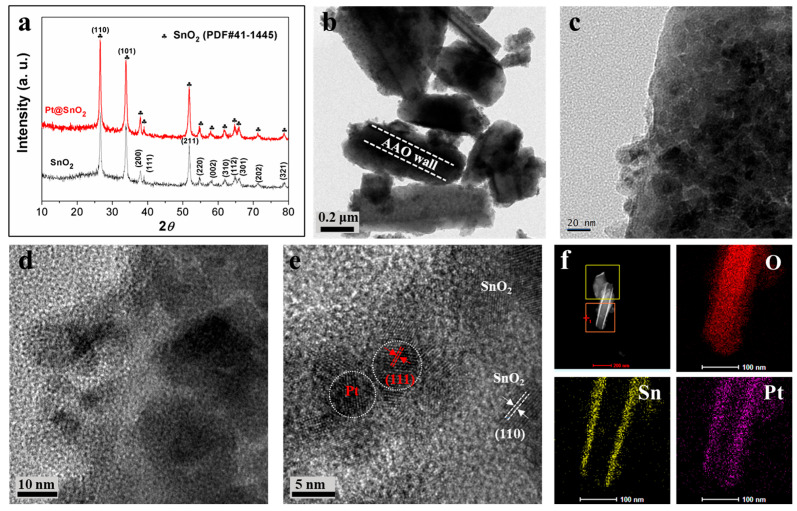
(**a**) XRD spectra of Pt/SnO_2_; (**b**,**c**) TEM and (**d**,**e**) HRTEM of Pt decorated SnO_2_; (**f**) element mapping of O, Sn, Pt of Pt/SnO_2_ nanotubes from the orange box.

Figure 3 provides a comprehensive overview of the crystal structure, morphology, and elemental distribution of Pt-modified SnO_2_ nanotubes, providing critical insights into the material’s synthesis and properties. In the XRD spectra (Figure 3a), the characteristic diffraction peaks of SnO_2_ (PDF#41-1445) are clearly identified, corresponding to its tetragonal rutile structure. Importantly, the Pt modification does not result in any observable shifts or new diffraction peaks in the XRD pattern. This indicates that the Pt modification does not significantly alter the crystal structure of SnO_2_. The absence of distinct Pt-related diffraction peaks can be attributed to the low Pt loading and its high dispersion, which are below the detection threshold of XRD. TEM images (Figure 3b,c) visually confirm the successful deposition of SnO_2_ on the AAO template. The SnO_2_ nanostructures adhere to the AAO walls, forming a well-defined architecture. Further structural details are revealed by HRTEM (Figure 3d,e). Clear lattice fringes are observed, with a spacing of 0.227 nm corresponding to the (111) plane of Pt and a spacing of 0.334 nm matching the (110) plane of SnO_2_. These distinct lattice signatures provide direct evidence for the successful synthesis of Pt-decorated SnO_2_, verifying the formation of a heterostructure, where Pt nanoparticles are anchored on the SnO_2_ matrix. Elemental mapping (Figure 3f) offers spatial distribution information of O, Sn, and Pt. The uniform presence of these elements across the nanotube structure further validates the successful incorporation of Pt into the SnO_2_ system. The overlapping and well-distributed elemental signals align with the structural characterizations, confirming that Pt is effectively modified on SnO_2_ and uniformly distributed within the composite.

### 3.2. Gas-Sensing Properties

**Figure 4:**
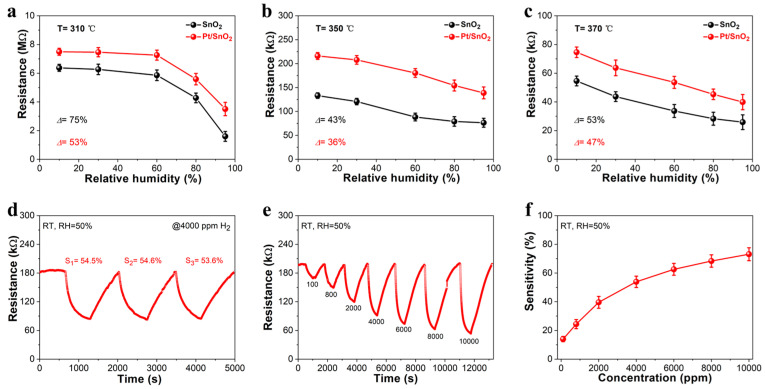
Humidity effect based on Pt/SnO_2_ sensors synthesized at (**a**) 310 °C; (**b**) 350 °C; (**c**) 370 °C; Gas-sensing properties based the optimized Pt/SnO_2_ sensor (350 °C): (**d**) Repeatability; (**e**) dynamic sensing curve for H_2_ detection at room temperature; (**f**) sensitivity vs. concentration.

Figure 4 systematically assesses the humidity impact and gas-sensing performance of Pt/SnO_2_ sensors fabricated via USP at 310 °C, 350 °C, and 370 °C, respectively. The humidity-resistance profiles (Figure 4a–c) evaluate how deposition temperature (consistent with the morphological variations in Figure 2) influences sensor performance under varying humidity. At 310 °C (Figure 4a), both SnO_2_ and Pt/SnO_2_ sensors exhibit high baseline resistance and significant resistance fluctuations (Δ = 53% for SnO_2_, Δ = 75% for Pt/SnO_2_) with increasing relative humidity (RH). This aligns with the porous, loosely packed nanostructure (Figure 2a,b), where moisture easily penetrates voids, disrupting electron transport. At 350 °C (Figure 4b), the Pt/SnO_2_ sensor shows a more stable resistance response, with a reduced resistance change (Δ = 36%) compared to 310 °C. The optimized particle packing (Figure 2c,d) minimizes moisture-induced disruptions: tighter interparticle contacts maintain electron conduction paths, while partial AAO pore retention balances gas access and humidity shielding. This structural optimization mitigates the effects of humidity through two key mechanisms. First, the denser SnO_2_ nanoparticles reduce the specific surface area exposed to water vapor, limiting the formation of adsorbed water layers. Second, the uniformly distributed Pt nanoparticles on SnO_2_ act as hydrophobic sites that repel the physical adsorption of water molecules, suppressing competition with oxygen for adsorption sites. Moreover, the moderate pore connectivity facilitates the rapid diffusion of water vapor, preventing the accumulation of condensed water within the channels. As a result, the density of adsorbed oxygen remains relatively stable across varying humidity levels, and the depletion layer thickness of SnO_2_ is less affected, leading to a stable baseline resistance. In comparison, for SnO_2_ (no Pt modification), resistance also decreases more gradually (Δ = 43%), but Pt/SnO_2_ outperforms in humidity stability—confirming Pt’s role in enhancing humidity tolerance. At 370 °C (Figure 4c), the Pt/SnO_2_ sensor again exhibits larger resistance swings (Δ = 47%), consistent with pore blockage (Figure 2e,f). Moisture accumulates in blocked pores, reintroducing instability—undermining the benefits of reduced resistance from larger particles. The blocked pores act as “traps” for water vapor, where moisture condenses into liquid droplets due to restricted evaporation. These droplets form conductive paths between adjacent SnO_2_ nanoparticles, short-circuiting electron transport and causing a sharp decrease in baseline resistance. Additionally, the larger SnO_2_ nanoparticles (100 nm) have fewer surface defects, weakening the chemical adsorption of oxygen, allowing water molecules to dominate the surface and further reducing the depletion layer, which lowers the resistance. The combined effect of condensed water and suppressed oxygen adsorption leads to larger resistance swings compared to the 350 °C sensor. Based on these results, the 350 °C deposition process was selected to fabricate the gas-sensing device for further performance evaluation. In addition, we conducted a comprehensive analysis to assess the impact of varying Pt loading on the gas-sensing performance of Pt/SnO_2_ sensors. Different Pt loading concentrations were achieved by maintaining continuous stirring for 3 h, 6 h, and 12 h, respectively. This clearly demonstrated that the Pt(6 h)/SnO_2_ sensor exhibits the highest sensitivity (53.9%@ 4000 ppm), significantly outperforming both Pt(3 h)/SnO_2_ (15.8%) and Pt(12 h)/SnO_2_ (34.9%). The improved performance of the Pt(6 h)/SnO_2_ sensor can be attributed to the optimal distribution of Pt nanoparticles on the SnO_2_ surface, which enhances electron transfer and facilitates the efficient adsorption of H_2_ molecules. In contrast, the Pt(3 h)/SnO_2_ sensor shows lower sensitivity, likely due to insufficient Pt loading that limits the number of active sites available for gas adsorption. The Pt(12 h)/SnO_2_ sensor, although it has a higher Pt loading, shows reduced sensitivity due to the agglomeration of Pt nanoparticles, which decreases the available surface area for gas interaction and impairs the sensor’s overall performance. Therefore, the optimal Pt loading achieved with 6 h of stirring results in the most efficient gas-sensing performance.

Under room temperature (RT, 25 °C) and fixed RH (50%), the Pt/SnO_2_ sensor (350 °C) undergoes three cycles of 4000 ppm H_2_ exposure. The resistance responses show minimal deviation: the sensitivities (S_1_ = 54.5%, S_2_ = 54.6%, S_3_ = 53.6%) are nearly identical, confirming excellent repeatability. This excellent repeatability is attributed to the stable nanostructure formed at 350 °C (Figure 2c,d), where uniform particle packing ensures consistent gas–particle interactions across cycles. The dynamic sensing curve (RT, RH = 50%) for H_2_ sensing from 100 to 10,000 ppm is shown in Figure 4e. The sensor exhibits rapid resistance modulation: upon H_2_ exposure, resistance drops (n-type SnO_2_ reacts with reducing gas), and it recovers when H_2_ is purged. The response/recovery times remain consistent across cycles, reflecting efficient gas diffusion (unblocked pores) and reversible surface reactions. The 350 °C-optimized structure balances diffusion kinetics (partial pore retention) and reaction sites (Pt-SnO_2_ heterojunctions), enabling reliable dynamic detection. Sensitivity increases with H_2_ concentration from 100 to 10,000 ppm, reaching ~80% at 10,000 ppm. The sensitivity and wide range confirm the sensor’s capability to detect both low-level (100 ppm) and high-level (10,000 ppm) H_2_. This performance is attributed to (1) Pt’s catalytic activity, which lowers H_2_ activation energy, and (2) the 350 °C-derived morphology—sufficient porosity for gas transport and dense packing for humidity resistance.

**Figure 5:**
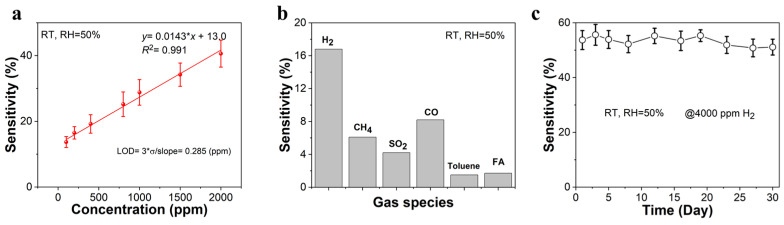
(**a**) The linear relationship of sensitivity vs. concentration; (**b**) selectivity towards gases at 100 ppm; (**c**) long-term stability for H_2_ sensing at room temperature.

Figure 5 thoroughly quantifies the detection limit (LOD) and long-term stability of the Pt/SnO_2_ sensor. In Figure 5a, a detailed sensitivity–concentration calibration curve for H_2_ detection (ranging from 100 to 2000 ppm) under ambient conditions (RT, 25 °C; RH = 50%) is illustrated. The linear regression analysis yields a calibration equation of (*y* = 0.0143 × *x* + 13.0), accompanied by an exceptionally high coefficient of determination (*R*^2^ = 0.991), which attests to the outstanding linearity and consistency of the sensor’s response within the specified concentration regime. The LOD is rigorously determined through the application of the statistical formula (LOD = 3 × σ/slope), where σ denotes the standard deviation of the baseline noise, and the slope represents the sensitivity of the calibration curve. This analytical approach results in a calculated LOD of 285 ppb, signifying the sensor’s remarkable ability to detect trace amounts of H_2_ [22]. This superior sensitivity can be attributed to the synergistic interplay between the optimized nanostructure (Figure 2c,d) and the Pt modification. The uniform particle packing of the nanostructure ensures efficient gas diffusion and electron transport, while the Pt-catalyzed surface reactions significantly lower the activation energy for H_2_ oxidation, thereby enhancing the sensor’s responsiveness to minute H_2_ concentrations even at low concentrations. Selectivity tests of the Pt/SnO_2_ sensor were conducted (Figure 5b), revealing that the sensor exhibits high sensitivity to H_2_ followed by CH_4_, SO_2_, and CO, toluene, and formaldehyde (FA) at 100 ppm. The long-term stability of the sensor is exhaustively evaluated in Figure 5c, where the sensor is continuously exposed to 4000 ppm of H_2_ under constant ambient conditions (RT, 25 °C; RH = 50%) over a period of 30 days. The sensitivity values exhibit negligible variations, remaining well within a narrow statistical range throughout the entire testing duration. These findings provide compelling evidence for the Pt/SnO_2_ sensor’s suitability for a wide array of practical applications, particularly those demanding high-sensitivity, ultralow-level detection, and long-term operational stability in H_2_ monitoring scenarios. To better contextualize the performance of our 3D Pt/SnO_2_ sensor, Table 1 presents a comparative analysis of its key parameters against those of state-of-the-art SnO_2_-based H_2_ sensors reported in the literature [23,24,25,26,27,28], highlighting the advancements offered by our novel 3D Pt/SnO_2_ design, especially in terms of achieving high sensitivity and low detection limits at room temperature.

### 3.3. Gas-Sensing Mechanism

**Figure 6:**
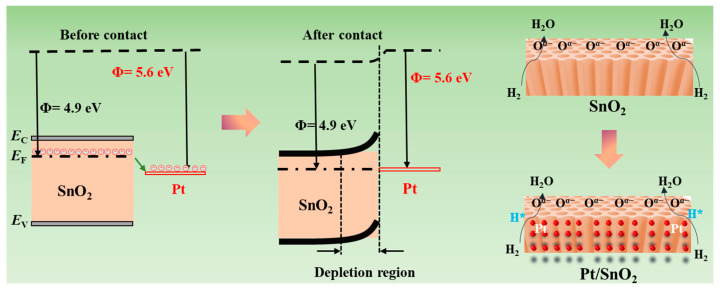
Possible sensing mechanism based on Pt/SnO_2_ for H_2_ sensing.

The sensing mechanism of the Pt/SnO_2_ sensor for H_2_ detection arises from synergistic interfacial and surface processes, as elucidated by the energy band structure and reaction dynamics in Figure 6. Before contact, SnO_2_ (work function, Φ = 4.9 eV) and Pt (Φ = 5.6 eV) have distinct work functions, maintaining independent equilibrium states-of-energy bands (conduction band *E*_C_, valence band *E*_V_, Fermi level *E*_F_ and electron distributions 23^)^. Upon contact, the work function disparity drives electron transfer from SnO_2_ to Pt, disrupting SnO_2_’s energy-band equilibrium and forming a depletion region at the Pt–SnO_2_ interface. In this region, free electron density is suppressed, altering SnO_2_’s electrical conductivity, resulting in the increased resistance shown in Figure 4a–c. In the clean air, oxygen would adsorb on the surface of the sensing material, capturing electrons from SnO_2_ and inducing the chemical oxygen adsorption. At room temperature, the dominant adsorbed oxygen species is O_2_^−^ (formed via O_2_(g) + e = O_2_^−^(ads)) due to its low activation energy, which further expands the depletion layer of SnO_2_. This process leads to the high resistance state ($R_{a}$). When exposed to H_2_, Pt functions as a catalytic site to dissociate H_2_ into atomic hydrogen H*. Pt lowers the activation energy for H_2_ dissociation, enabling the efficient cleavage of H-H bonds even at room temperature. These H* species diffuse to the Pt–SnO_2_ interface, reacting with pre-adsorbed O_2_^−^ via the redox reaction—H*(ads) + O_2_^−^(ads) = H_2_O(g) + e^−^—and releasing electrons back into SnO_2_, narrowing the depletion layer and increasing free electron concentration, causing the low resistance state ($R_{a}$). Thus, the great change in the resistance caused the improvement of the sensitivity ($=(R_{a}-R_{g})/R_{a}*100\%$). Additionally, the 3D nanostructure (3D AAO template-supported SnO_2_ nanotubes) enhances sensing performance by providing a high surface-to-volume ratio (abundant active sites) and interconnected porous channels (accelerating H_2_ diffusion and H_2_O desorption), which amplifies the resistance change and shortens response/recovery times. In essence, the Pt/SnO_2_ heterostructure enhances H_2_ sensing via two key effects: (1) work function-driven electron transfer, which establishes the depletion region, and (2) Pt-catalyzed H_2_ dissociation, which modulates the depletion region via surface reactions. The coupling of these processes enables the transduction of H_2_ presence into measurable electrical signals, underpinning the sensor’s high sensitivity and selectivity for practical gas-sensing applications.

## 4. Conclusions

In this study, a 3D SnO_2_-based gas sensor was successfully developed to overcome the challenges of high operating temperature and limited sensitivity typically associated with traditional SnO_2_-based sensors for H_2_ detection. The sensor was fabricated by depositing SnO_2_ nanoparticles onto a 3D AAO template using ultrasonic spray pyrolysis, followed by modification with Pt nanoparticles. This design enables efficient H_2_ detection at room temperature. Characterization results revealed that SnO_2_ nanoparticles formed a uniform and dense structure on the AAO template at 350 °C, providing the sensor with excellent responsiveness to H_2_ in the concentration range of 100–10,000 ppm, with a low detection limit of 285 ppb, and remarkable long-term stability. The sensing mechanism is based on the formation of a depletion layer at the Pt/SnO_2_ interface due to electron transfer, where Pt catalyzes the dissociation of H_2_ into atomic hydrogen (H*). These H* species then react with surface oxygen species, attributed to the sensing performance improvement. This study presents an effective solution to overcome the high-temperature limitations of traditional gas sensors, offering significant potential for H_2_ energy safety monitoring.

## Figures and Tables

**Table 1:** State-of-the-art SnO_2_-based H_2_ sensor comparison.

Materials	Tem. (°C)	H_2_ Con. (ppm)	Response	LOD ^c^	Ref.
3D Pt/SnO_2_ nanotubes	RT	100	16.5% ^a^	285 ppb	This work
CeO_2_-doped SnO_2_	160	50	23.7 ^b^	10 ppm	[23]
Pd-doped rGO/ZnO–SnO_2_	380	100	9.4 ^b^	50 ppb	[24]
Ce-doped SLG/SnO_2_	250	10	2.49 ^b^	500 ppb	[25]
Pd@MoS_2_	RT ^d^	15,000	4.44 ^b^	300 ppm	[26]
In-In_2_O_3_	RT ^d^	1500	3.4 ^b^	/	[27]

^a^ Response = (R_a_ − R_g_)/R_a_ × 100%; ^b^ Response = R_a_/*R*_g_; ^c^ LOD = limit of detection, ^d^ RT: Room temperature.

## Data Availability

The authors will supply the relevant data in response to reasonable requests.
